# Developing a Virtual Reality Educational Tool to Stimulate Emotions for Learning: Focus Group Study

**DOI:** 10.2196/41829

**Published:** 2023-03-20

**Authors:** Silje Stangeland Lie, Kari Røykenes, Aleksandra Sæheim, Karen Synne Groven

**Affiliations:** 1 Faculty of Health Studies VID Specialized University Stavanger Norway; 2 Faculty of Health Studies VID Specialized University Bergen Norway; 3 Faculty of Social Sciences VID Specialized University Oslo Norway; 4 Section for teaching and learning Østfold University College Fredrikstad Norway; 5 Faculty of Health Oslo Metropolitan University Oslo Norway

**Keywords:** virtual reality, 360° video, learning, experiences, emotions, health care and social work higher education

## Abstract

**Background:**

By watching 360° videos in virtual reality headsets, students may experience being immersed in the portrayed situation. There is a paucity of empirical studies on the application of immersive 360° videos watched in virtual reality headsets for students in health care and social work education and the pedagogical theory guiding the development of such educational tools. This led to our interest in exploring how a virtual reality educational tool involving 360° videos can stimulate emotions and how this can be used as a pedagogical tool in these educational programs.

**Objective:**

The aim of this study was to explore the experiences of faculty members and students regarding a prototype 360° video watched in virtual reality headsets during the development phase of an educational project. We addressed the following research questions: *How does the virtual reality prototype stimulate emotions? How can virtual reality be used in higher education for health care and social work students?*

**Methods:**

We used a qualitative design and collected data through focus group interviews with project participants. The data were analyzed using qualitative content analysis.

**Results:**

Our analysis identified 2 main themes in participants’ experiences with the virtual reality prototype. The first theme highlights that when participants experienced watching the 360° video in a virtual reality headset, it stimulated their emotions as an *authentic professional experience* would. The second theme, *contextualization of virtual reality*, highlights participants’ perceptions of how the virtual reality experience should be incorporated into a safe educational context.

**Conclusions:**

Our findings suggest that 360° videos with human actors who use eye contact with the camera can trigger emotions in the viewer and therefore serve as a pedagogic tool that can create authentic professional experiences for students. The participants expressed the view that the virtual reality educational tool could be used to prepare students for real-life practice in health care and social work. However, they underlined that 360° videos in virtual reality need to be contextualized in educational programs to create a safe environment for learning and to ensure follow-up on the emotions such experiences can trigger in students. Our results highlight the perceived importance of allowing students to reflect on the virtual reality experience in a safe setting and of follow-up by faculty members. In-person follow-up with students can be resource intensive for programs with large numbers of students and makes it challenging to offer repeated training, something that has been identified as one of the benefits of virtual reality.

## Introduction

### Background

Virtual reality can be defined as a digital representation of a 3D environment [1]. The current general understanding of virtual reality involves the use of head-mounted displays to create a fully immersive experience [2,3]. When watching 360° videos in virtual reality headsets, students may experience being immersed in the situation portrayed, although they cannot act in it. There is a paucity of empirical studies on the application of 360° videos watched in virtual reality headsets in health care and social work education and the pedagogical theory guiding the development of such educational tools [4]. This led to our interest in exploring how a virtual reality educational tool involving 360° videos can stimulate emotions and how this can be used as a pedagogical tool in these educational programs.

In health care and social work higher education, virtual reality may allow students to experience, practice, and master dealing with challenging situations without putting clients, patients, or themselves at risk of harm [5-7]. Hence, virtual reality can serve as a valuable didactic tool in higher education, where students are expected to learn how to deal professionally with challenging situations, such as complex interactions with patients or clients considered vulnerable. The use of virtual reality in health care and social work higher education is still new [4,6]. Although recent studies show that it is gaining ground in simulation training for clinical skills and role-play in medical education [8], there is still a research gap on the use of virtual reality to facilitate nontechnical and soft skills in health care and social work higher education [9]. Most studies on the use of virtual reality in higher education explore experimental projects, such as prototyping and testing with students [6,10,11].

By the time they complete higher education, health care and social work students are expected to master complex, challenging interactions with patients or clients. Until now, it has been difficult to train these skills outside of field placements, and this is our main rationale for using virtual reality for skills training in health care and social work higher education. Previous studies suggest the need for future research to investigate alternatives to computer graphics to increase the realism of virtual reality [1]. One such alternative is spherical immersive (360°) video. The use of 360° video in virtual reality entails considerable potential for clinical medical training as well as for health care and social work education [4,8]. Using virtual reality for training soft skills is a new approach [6,10], but it has potential to prepare students for professional practice. However, more research is needed on how 360° videos may influence emotions and empathy for the viewer [8], and there is a call for more research on using 360° videos as pedagogical activities for learning in health care and social work higher education [4].

Engaging, authentic learning tasks increase students’ motivation and can give them a sense of mastery [12]. Currently, most studies of educational technology focus primarily on the technology itself, paying too little attention to pedagogy and learning design [4], and there is still a gap between the institutional rhetoric around the use of technology in education and the reality of its use [13]. Radianti et al [10] recommend that future educational virtual reality applications be thoroughly explored using qualitative research methods to assess students’ knowledge, skills, and learning experiences. The use of virtual reality to reach competency goals may require adjustments to practice and a new understanding of simulation and skills training. Existing research on the use of virtual reality to train students in communication, collaboration, soft skills, and behavioral impacts is too limited to offer any practical recommendations for design or use [10]. Moreover, most existing research on the use of virtual reality in higher education fails to draw on specific learning theories [4,10].

### Theoretical Considerations

The theory of experiential learning developed by Kolb [14] proposes a model of the learning process that emphasizes the importance of experience in the development of knowledge and skills. According to Kolb [14], learning is a continuous and ongoing process that involves the acquisition and transformation of knowledge through experience. The learning process consists of 4 stages: concrete experiences, reflective observation, abstract conceptualization, and active experimentation. Concrete experiences refer to the students’ firsthand experiences, such as participating in a hands-on activity or observing an event. Reflective observation involves thinking about and analyzing the individual’s experiences to identify patterns and meaning. Abstract conceptualization involves developing theories, principles, and concepts based on the patterns and meaning identified through reflective observation. Active experimentation involves applying the theories, principles, and concepts developed through abstract conceptualization to new situations to test their validity and usefulness [14]. According to Kolb [14], learning occurs when an individual moves through all 4 stages of the learning process and integrates their experiences into their existing knowledge and skills. The learning process is social and ongoing and involves the continual cycle of experiencing, reflecting, conceptualizing, and experimenting. Accordingly, higher education should be organized around this cycle. It begins with a concrete experience, followed by reflection—either alone or with peers—to develop new knowledge. Next, new concepts and theories are introduced to give meaning to the experience, and, finally, new action is taken to test the student’s expanded knowledge and gain new experience. This enables students to reflect and implement well-considered actions in new situations [14].

Emotions are complex psychological and physiological responses to experiences that are accompanied by various behavioral and physiological changes [12]. Emotions may influence learning by affecting cognitive resources, long-term memory, cognitive and metacognitive thinking strategies, and motivation [15-17]. According to Sinding and Stiegler [18], humans have primary emotions as well as secondary emotions. Primary emotions are also called basic emotions, which are direct reactions to a situation. Secondary emotions are emotional responses to a basic emotion. Researchers have differing opinions on the number of human emotions and on how they should be described. However, many researchers focus on 7 basic emotions: “joy, sadness, fear and anxiety, anger, shame, guilt, and disgust” [18]. Emotions have a strong influence on attention, and emotional events are remembered longer and more vividly. Moreover, emotions can lead to better problem-solving in education, and they can play a key role in engagement with, and completion of, academic tasks [15,19].

According to Pekrun [12], positive emotions during learning, such as positive expectancy emotions, trigger students’ interest and motivation and therefore support learning. However, negative emotions can also sometimes improve learning and achievement. This is because such emotions may lead students to focus and apply themselves [12]. Although basic emotions such as anger and anxiety (as well as secondary emotions such as stress, insecurity, and irritability) [18] generally cause barriers to learning, these emotions may also activate learning. This is because they may sharpen students’ attention, which can support their achievement of learning outcomes [20]. According to the circumplex model of affect developed by Russell [21], emotions are classified into 2 categories: valence and arousal. Valence is the experience of a situation as either positive or negative. Arousal is the experience of physiological activation or deactivation in a situation. High arousal activates attention and memory, whereas valence has a more complex impact on learning. Positive experiences may increase attention and reinforce cognitive processes, but negative experiences may also promote deeper analysis and sharpen detailed memory [22].

Watching 360° videos in virtual reality headsets demands monotasking and therefore facilitates complete focus on the video. This is called immersive virtual reality [4]. Earlier research has found that 360° videos as well as virtual reality have the potential to generate experiences and create emotional reactions in the viewer [4,7]. We can assume that these emotional reactions may induce engagement and deeper cognitive processing, which could then facilitate learning [4,7,14,19,23]. Developers of educational content must be aware of the strong influence of emotions on memory and learning.

### Aim and Research Questions

There is little knowledge of how virtual reality stimulates emotions and thereby learning in higher education for health care and social work professionals. To address this gap in the literature, we conducted a qualitative study as part of the development phase of an educational development project. In this project, 360° videos were developed for a training program aimed at bachelor’s degree (undergraduate) students in nursing, occupational therapy, social education, and social work. These videos are designed to be viewed in virtual reality headsets. Hence, the aim of this study was to explore the experiences of faculty members and students regarding a prototype 360° video viewed in virtual reality headsets during the development phase of an educational project. We addressed the following research questions:

- How does the virtual reality prototype stimulate participants’ emotions?
- How can virtual reality be used in higher education for health care and social work students?

## Methods

### Research Design

We used a qualitative design and collected data through focus group interviews with project participants. Qualitative research is useful for exploring participants’ experiences, including emotions related to concrete phenomena. This approach was therefore chosen as the method for this explorative study.

### Context

#### Development

This study was undertaken as part of a larger ongoing project that aimed to develop, test, and evaluate a virtual reality educational tool with 360° videos to increase student activity in higher education. This tool is designed for, and was tested with, bachelor’s degree students in nursing, social education, occupational therapy, and social work at a higher education institution in Norway in the academic year 2022-2023. The project focuses on user participation and involvement to develop an effective virtual reality educational tool. Initial user involvement is described in the next section.

#### Initial User Involvement

During the spring semester of 2021, a total of 130 students in their final year of a bachelor’s degree course in social work conducted projects in quantitative methods. They were assigned a topic and asked to develop a questionnaire, analyze the data, and present their findings. Three student groups were assigned topics that could help the project group developing the 360° videos to understand what kind of virtual reality scenarios would interest students. These 3 student groups chose the focus and wording of their survey questions. One group asked respondents to choose among different scenarios, whereas the other 2 groups focused on emotions and reactions. This focus on emotions and reactions gave the project group insights into students’ perspectives on the best focus for virtual reality scenarios. To increase student participation during the development phase of the project, the project group developed an anonymous electronic questionnaire based on the surveys from the student project. First, social work students responded to this questionnaire; later, the questionnaire was sent to associate professors and university teachers as well as second- and third-year students in all 4 target disciplines (nursing, social education, occupational therapy, and social work) and representatives from clinical practice.

We received 232 responses to the questionnaire (n=114, 49.1% students; n=87, 37.5% representatives from clinical practice; and n=31, 13.4% associate professors and university teachers), and the virtual reality educational tool was developed based on responses to the survey. The results are described in the next section. A key point is the students’ desire to practice situations that could trigger high emotional arousal and discomfort.

#### Results of the Mapping Questionnaire

The survey is provided in Multimedia Appendix 1 [18]. The students (n=114) who responded to the survey represented all 4 disciplines involved. Of these 114 students, 34 (29.8%) were nursing students, 23 (20.2%) were social education students, 21 (18.4%) were occupational therapy students, and 36 (31.6%) were social work students. The respondents were asked to rate fields of work that they thought students needed more experience with during their education. The students ranked mental disorders as the first priority and placed drug addiction second. The associate professors and university teachers who responded ranked mental disorders equally with interdisciplinary collaboration. The representatives from clinical practice ranked cognitive disabilities as the first priority and placed interdisciplinary collaboration second. The respondents were also asked to rate which client or patient emotions the students would benefit from encountering in a VR session. We were also interested in identifying skills that students need to practice more. Student responses to this question were unanimous: they wanted more practical training in managing conflicts, setting boundaries, and prevention and use of coercion. All representatives from clinical practice (n=87) as well as associate professors and university teachers (n=31) identified building trust as the most important skill. They also rated communications skills and ethical reflection higher than students did on this question.

#### Virtual Reality Educational Tool With 360° Videos

On the basis of the questionnaire responses, we developed scripts for challenging scenarios that were designed to trigger students’ emotions. By allowing students to experience these stressful scenarios through virtual reality headsets, we aimed to engage the students in deeper cognitive processes in a safe environment. As this learning cycle began with immersive virtual reality, it can be said that the starting point for learning was a concrete experience. The scenario portrayed in the prototype video was filmed with a 360° camera at a health care institution to secure realism. Three amateur actors played out the scene. The scenario script is described in the next section.

#### The Script for the Prototype Scenario Ivar

A man, Ivar, sits in a wheelchair in his room at the institution Solstien 3. He is sitting at the dining table with a full dinner plate in front of him. The viewer (a student) is sitting at the table with Ivar. A professional caregiver enters the room, talks softly to Ivar, and comments that he has not eaten anything and that he looks tired.

Suddenly, Ivar’s sister bursts into the room, talking loudly and quickly. She claims that Ivar needs to be more active so that he can rehabilitate from the stroke that put him in the wheelchair. The professional tells her that Ivar is about to rest.

The sister persists. She wants Ivar to go for a walk with her. She tells the professional to help him get dressed for a walk. The professional tries to ask Ivar what he wants, but Ivar does not respond. They both look at the viewer, and the scene fades into an interactive choice:

- Option 1: agree with the sister and find Ivar’s clothes.
- Option 2: ask Ivar again if he would like to join his sister for a walk.
- Option 3: firmly tell the sister that Ivar does not want to take a walk.
- Option 4: suggest that Ivar and his sister have a coffee together instead.

The scene then plays out in 4 different ways depending on the user’s choice (ranging from Ivar angrily throwing his dinner plate on the floor to Ivar sitting in his chair, apathetic and unresponsive).

During a project workshop, the prototype 360° video was watched by all participants using Oculus Quest 2 virtual reality headsets (Meta). The software used to display the 360° video and provide the interactivity was CenarioVR (ELB Learning) [24].

### Recruitment and Participants

Twelve participants (n=9, 75% faculty members and n=3, 25% students) were recruited from the project group for the focus group interviews after their participation in the workshop. The first author invited all members of the project group verbally as well as in writing. At the time of data collection, the project group included 18 people (n=13, 72% faculty members and n=5, 28% students in the bachelor’s degree programs in nursing, social education, occupational therapy, and social work). Before the interviews, the project group members had worked together to develop the video scripts and the learning activities based on the initial survey responses, as described previously.

### Data Collection

Data were collected through 3 focus group interviews held in September 2021. The first focus group interview was conducted in person at a meeting room and included 4 of the 9 faculty members and 1 of the 3 students. The second focus group interview was conducted on the web and included 3 of the 9 faculty members and 1 of the 3 students. Finally, the third focus group interview was conducted on the web and included 2 of the 9 faculty members and 1 of the 3 students. The mix of in-person and web-based interviews was necessary because the COVID-19 pandemic (2020-2022) hindered traveling and meeting in larger groups. Furthermore, remote interviews require less time and energy than face-to-face interviews [25]. Web-based meetings were therefore considered appropriate both practically and methodologically for the project group participants located in a city different from that in which the facilitator resided.

The first author, an experienced qualitative researcher, performed all 3 focus group interviews using a semistructured interview guide. This method of data collection is characterized by a nondirective style of interviewing that encourages discussion and the expression of a variety of viewpoints [26]. Topics were introduced and discussed based on the participants’ experiences and opinions. The participants were encouraged to speak freely, and the facilitator encouraged topical discussions among the participants in each focus group. The topics addressed were as follows: (1) the participants’ expectations of virtual reality, (2) their experience with the prototype 360° video, (3) how the action and story in the prototype 360° video engaged them, (4) how virtual reality and 360° videos could be implemented in their education, and (5) what kind of learning activities are promoted by immersive virtual reality. The interviews lasted 60 to 70 minutes. All interviews were audio recorded and subsequently transcribed verbatim. To strengthen the reliability of the data collection, the first author conducted all 3 interviews, and all group discussions were guided by the same 5 aforementioned predefined topics.

After the initial data analysis, the first author asked 3 faculty members and 1 student to comment on the transcripts to gain more information about some of the topics that were discussed in the interviews. This is in accordance with methods in the literature on conducting qualitative interviews [26,27]. The comments concerned the emotions the participants experienced while watching the virtual reality video, the development of student activities related to the scenario, and how instructors can follow up on the emotions that students experience during such learning activities.

### Data Analysis

The transcribed interview data were subjected to qualitative content analysis as described by Graneheim and Lundman [28]. As the aim of this study was to explore participants’ experiences, this versatile method, which allows the analyzer to choose the level of abstraction and interpretation, was deemed appropriate [29].

First, the authors read all the interview transcriptions to obtain a comprehensive understanding of the data. Meaning units related to the aim of the study were identified and shortened, preserving their core content. These condensed meaning units were then labeled with tentative codes. Thereafter, categories were created by comparing and grouping codes according to similarities and differences. Next, to strengthen the reliability of the analysis, the research team discussed and revised the codes and categories several times until a consensus was reached. The contents of the final categories were abstracted into 2 main themes, which were also discussed, renamed, and considered many times before they were finalized.

To strengthen the trustworthiness of the data analysis, the findings and interpretations were discussed by all authors to strengthen the reliability of the analysis. The authors come from different backgrounds in health care and social work (nursing, physiotherapy, and social work), and all are experienced researchers. We worked for transparency of the analysis process by following the steps described by the COREQ (Consolidated Criteria for Reporting Qualitative Studies) 32-item checklist (Multimedia Appendix 2 [27]).

### Ethics Approval, Informed Consent, and Participation

This project is registered with the Norwegian Agency for Shared Services in Education and Research (423788), and all data were collected and stored according to its guidelines. Before the data collection, all informants signed informed consent forms. They were informed that they could withdraw from the study at any time, without any negative consequences. The data were stored securely and deidentified. No compensation was given to the participants.

## Results

### Main Themes

In the analysis, we identified 2 main themes in participants’ experiences with the virtual reality prototype. The first theme, *authentic professional experience*, highlights participants’ experiences of watching the 360° video in a virtual reality headset. The second theme, *contextualization of virtual reality*, highlights participants’ perceptions of how the virtual reality experience should be incorporated into the educational context*.* Each of these themes includes 2 categories, as shown in Textbox 1.

**Overview of themes and categories.**

Authentic professional experience

- Involvement in the situation
- Practicing complex, realistic situations

Contextualization of virtual reality

- Exploring emotions
- Stimulating reflection

Our findings are organized around these 2 main themes. In the following sections, illustrative quotations from the interviews are included to increase transparency.

### Authentic Professional Experience

While using the virtual reality headset, participants felt as though they were present in the scenario. This sense of participation had an emotional impact on the participants. Both students and faculty members stated that immersion in the challenging situation portrayed in the film created a sense of authenticity and gave them a genuine professional experience.

Participants reported an experience of genuine *involvement in the situation.* When the actors looked directly at the camera to mimic eye contact, participants felt that they were expected to answer or respond. This use of eye contact made participants feel close to the situation, activating a range of emotions. Participants mentioned not only empathic emotions but also feelings of frustration: they were unsure how or whether they should or could react. They found this sense of involvement both interesting and frustrating. At the same time, participants said that watching the situation through the immersive virtual reality headset made them feel involved in it. Virtual reality made the situation feel close, involving emotional engagement that activated reflection on how to respond. This is illustrated in the following quotation:

I was quite surprised at how many emotions I felt when I saw the video. One of the actors often looked at the camera and it felt like she expected me, the person wearing the virtual reality goggles, to answer her or respond some way. It felt close and made me think.

Faculty member

The participants found that watching the 360° video through a virtual reality headset enabled them to practice *complex, realistic situations* in a safe environment. The participants compared this experience to reading about a patient case. One student highlighted their sense of being involved in a realistic situation: “It became more realistic, more genuine.” The participants also said that the immersive encounter created a unique mutual experience for the viewers:

They all have this same experience: they have seen the same video, and that is pretty unique. Instead of hearing a situation retold by others, they have all had the experience.

Faculty member

Some challenges of the portrayed scenario, particularly concerning the sense of realism, was also mentioned. One suggestion for improvement involved the viewers’ placement in the room (ie, where the 360° camera was placed during filming). In our video, the camera was placed on a tabletop right in front of one of the actors, as though the viewer were sitting at the table and having a meal with the actor. Some of the participants felt that this camera placement was an unnatural one for a student, especially because the observer was silent throughout the scene. This created a feeling of incongruence that negatively affected the realism of the scenario. The participants felt that it would be important to consider camera placement more carefully when filming future scenarios to increase the sense of participation and realism.

### Contextualization of Virtual Reality

Students and faculty members also highlighted the importance of follow-up and creating a safe learning environment for students who watched the challenging scenario. They suggested that group discussions be held after the virtual reality experience, saying that the emotional impact of the scenario created both a need for, and motivation to engage in, subsequent learning activities. This suggestion emphasizes the importance of contextualizing the virtual reality experience.

The participants underlined the importance of *exploring the emotions* triggered by the scenario. They pointed out that emotions alone are not sufficient to promote learning. Students needed to explore their own emotions and the impact these emotions could have on future situations. Both students and faculty members underlined the need for the participants to meet and discuss the scenario in person in a safe learning environment, as illustrated in the following quotation:

To follow up on students’ emotions, we should facilitate a kind of debriefing after they watch the virtual reality scenario—to blow off steam. During this debriefing, it is important that we encourage them to focus on what they experienced, as it could quickly become just a reiteration of what they observed. It is important that they focus on their own emotions in this round. This should be clarified in advance.

Faculty member

Because of the importance of dialogue and personal follow-up, the participants felt that students should not individually watch the 360° videos in virtual reality headsets for self-training. They also pointed out that learning happens during later reflection and debriefing, not while a student experiences the situation in virtual reality. The participants underlined the central role of *stimulating reflection* after the virtual reality experience in a contextualized learning activity. This is illustrated in a quotation from a faculty member:

It’s not what happens inside the virtual reality goggles that facilitates learning. It’s what follows this activity, what you do afterwards. We cannot use virtual reality in isolation; we must look at the whole concept, the learning concept that we create. It’s during the following tasks and assignments that learning happens, not inside the virtual reality. I think that what happens within the virtual reality can create and evoke emotions. And through these emotions, a commitment to reflect on what happened is created. How can we face this? How do I understand this?

Faculty member

One student formulated their increased motivation to engage in subsequent learning activities, such as reflection, in this way: “The scenario gave us an extra push to learn, making us more engaged.” The participants also perceived that educational virtual reality activities could create fruitful opportunities for engaging students as peer facilitators, enabling learning for both parties. However, they pointed out the need to provide students with guidelines for the scenarios in advance for these to function effectively as learning activities. Group discussions to enable reflection and to find ways to handle the scenario professionally were frequently recommended. The participants also mentioned that group discussions could create space for interdisciplinary learning:

I think that would be a great idea: to gather people from different professions and then sit and discuss how we could handle the case in the scenario. To find the best solution for the patient, the sister, and the professional.

Student

## Discussion

### Principal Findings

This study provides insights into the development and use of a virtual reality tool in a higher education setting for health care and social work. Our findings illustrate that the use of 360° videos involving human actors and realistic professional scenarios can stimulate intense emotions in viewers. At the same time, our findings underline the need to contextualize the virtual reality experience in health care and social work higher education programs to help students manage these emotions and to facilitate learning in a safe setting.

### Comparison With Prior Work

Our findings show that participants found the virtual reality prototype to be realistic and immersive. In contrast, in previous studies, students have reported that virtual reality environments were insufficiently realistic and that this lack of realism limited their sense of immersion and inhibited the overall learning experience [1]. In our project, the 360° video was filmed with human actors. Similarly, Beverly et al [8] found that when virtual reality videos were used to train health care personnel, learners connected emotionally to the characters in the video. This in turn increased their empathy for the characters [8]. Another study reports that the use of human actors in virtual reality increases emotional engagement in learning [30]. Our findings, combined with these previous findings [1,8,30], build an argument that it is worthwhile to use human actors in 360° virtual reality videos for health care and social work higher education programs. Viewers can identify with characters portrayed by human actors more easily than with those portrayed by avatars. Interestingly, our findings also indicate that viewers experienced closeness and involvement in the situation portrayed because of the actors’ use of eye contact with the camera. This is a novel finding and could be presented as a recommendation for future 360° virtual reality videos.

Our findings show that unlike reading about cases or watching videos on a flat screen, watching a 360° video in a virtual reality headset gives the viewer a firsthand perspective on a concrete experience. This perspective provides students a unique opportunity to practice complex situations, something they are rarely able to do sufficiently during their training. It is both practically difficult and ethically questionable to allow students to encounter such situations in clinical placements, and students have previously identified this as a challenge in their training [31]. Virtual reality can therefore provide a safe learning environment for practicing such challenging scenarios. The participants felt involved in the portrayed situation, which triggered emotional reactions. It is therefore worthwhile to explore the implications of this sense of emotional arousal for the students’ learning processes. Our focus on developing scenarios designed to trigger intense emotions in the viewer was based on the mapping we conducted at the beginning of the project, described in the Context section of this paper. A key point in our mapping was students’ perceived need to practice situations that triggered high emotional arousal and negative emotions. Our findings indicate that the portrayed scenario and the emotions it triggers can increase students’ motivation to learn. As Kolb [14] points out, the entire being is involved in thinking, feeling, perceiving, and behaving during the learning process. Combined with experience, abstract thinking, and action, reflection contributes to a holistic learning process [14]. Thus, concrete experiences, such as those created by our virtual reality prototype, play a key role in activating reflection. Such reflection is central to the learning process because it can introduce new knowledge and give meaning to experience and enable the learner to implement well-considered actions in new situations [14]. Our participants viewed the immersive virtual reality experience as an authentic professional experience. This suggests ample possibilities for the educational use of virtual reality based on the sociocultural approach to learning. Our findings also support earlier evidence for the potential of virtual reality applications in higher education to improve learning and knowledge acquisition [6,8,11]. By facilitating learning through a virtual reality educational tool, we might better prepare students for practice in a safe setting.

However, our findings also underline the importance of contextualizing educational virtual reality experiences to facilitate a safe learning environment and enable students to reflect in ways that lead to knowledgeable action. Viewing 360° videos can engage students, but so may watching movies on Netflix. On the basis of the theory of experiential learning formulated by Kolb [14], which aligns with our findings, we find it valuable to create learning situations that activate learning by creating feelings of discomfort in a safe setting. Konow Lund et al [31] found that many nursing students experience emotionally challenging situations during their first clinical placement. These situations can trigger intense feelings, which the students may struggle to handle [31]. The authors argue for the importance of skilled supervision to help students develop “mature empathy” and the need for students to learn to adapt to the considerable demands they will face as professionals [31]. Our findings on the importance of student reflection on challenging scenarios in a safe setting and of follow-up by faculty members contribute to existing evidence on the use of virtual reality in health care and social work higher education.

Our findings show that viewing a 360° video in a virtual reality headset encouraged reflection. In our context, the scenario was constructed, and actors portrayed specific roles and situations according to a script. All participants experienced the same portrayed situation. This gave students a unique opportunity to discuss the experience in groups and construct knowledge together, building on each other’s experiences and theoretical knowledge, allowing all to participate and use their previous knowledge and experiences to analyze the situation in a safe setting. It enabled safe explorations of participants’ emotions. Negative emotions characterized by high arousal, such as fear and anger, may promote deeper analytic processes and sharpen detailed memory [19,32]. In our study, faculty members in particular underlined that learning happens during reflection and debriefing after the virtual reality experience, not while students are using the virtual reality headset. Thus, our participants argued that the reflections and discussions after the students watched the 360° video were the most important part of the learning process. This line of argument tallies with the notion expressed by Kolb [14] that experience alone is not sufficient; it must be combined with reflection. Facilitated reflection might minimize the risk that students might assume that a given scenario shows a single right or wrong way to handle a portrayed situation. Debriefing problematic situations and emotions supports learning and helps students to build knowledge and competence, and it may help them to process potentially conflicting or challenging emotions.

Nevertheless, it is important to note that emotions do not always have a positive influence on learning [19]. One should consider whether students’ use of 360° videos that portray challenging scenarios must be accompanied by in-person meetings with faculty members (eg, in groups, as suggested by students in the interviews). In-person follow-up may be extremely resource intensive, especially for programs with large student bodies, and does not facilitate repeated training, something that has been identified as a benefit of virtual reality in earlier studies [33,34]. This could hinder the use of virtual reality in health care and social work higher education. There is a need to further explore students’ perspectives on the need for in-person follow-up.

### Limitations

Our findings may serve as a basis for further work with virtual reality educational tools and for future research that aims to expand our understanding of the pedagogy of virtual reality educational tools. However, generalizations from this small situational study are neither possible nor intended. We have explored experiences with 1 specific prototype 360° video and the participants’ opinions of how this can be used in health care and social work higher education. Data collection was conducted by focus group interviews guided by a semistructured interview guide. Focus group interviews allow participants to describe their genuine experiences by conversing in a structured dialogue, providing rich data. For researchers, the aim of focus group interviews is to gather experiences and opinions; the aim is not to obtain objective data. Data collection and analysis are also dependent on the researchers’ preconceptions. This is a prerequisite in qualitative studies, described in the methods literature [26,35]. All informants (faculty members and students) were participants in the project group and were familiar with each other and with the interviewer.

One limitation is that the participants were involved in developing the script for the 360° video and may therefore have been invested in reporting positive experiences with the tool. We were aware of this challenge during the interviews, and the informants were therefore encouraged to reflect critically and describe challenges. Another limitation is that each focus group included only 1 student; most of the interview participants were faculty members. It would have been preferable to include more students in the testing as well as the interviews. However, this was impossible owing to the COVID-19 pandemic restrictions. Having more than 1 student in each interview might have strengthened the student point of view in our findings. We could also have interviewed all participating students in 1 focus group. However, this would have prevented discussions between the 2 parties of interest in the project (students and faculty members). Therefore, we decided to include 1 student in each focus group [35]. A third limitation is that the students involved in this study were also members of the project group as student participants. They might therefore have been particularly interested in virtual reality. Finally, a fourth limitation concerns the fact that the participants watched only 1 prototype 360° video. This may have limited the emotional palette explored with it, influencing the findings of this study. Although it could have been of value to watch several 360° videos, this study was undertaken during the development process of a larger study, and we only had the 1 video available at the time.

### Conclusions

Our findings suggest that 360° videos with human actors who use eye contact with the camera trigger emotions for the viewer. Such videos can therefore serve as pedagogical tools that may create authentic professional experiences for students. Using this as an educational tool can better prepare students for practice in a safe setting. However, our findings indicate that virtual reality experiences need to be contextualized within educational programs to create a safe learning environment and to ensure follow-up on students’ emotions. Our findings highlight the perceived importance of subsequent reflection in a safe setting and follow-up by faculty members. In-person follow-up with students can be resource intensive, especially for programs with large numbers of students, and does not facilitate repeated training, something that has been identified as a benefit of virtual reality.

## Appendix

Multimedia Appendix 1 Questionnaire mapping students’ training needs (context).

Multimedia Appendix 2 COREQ (Consolidated Criteria for Reporting Qualitative Studies) 32-item checklist.

## Abbreviations

COREQ: Consolidated Criteria for Reporting Qualitative Studies

## Data Availability

Because of ethical concerns, the data have restricted access, as emphasized through the participants’ informed consent form. Moreover, the data comprise transcribed interviews in Norwegian. Nevertheless, the data can be obtained by contacting the first author. However, access to the data depends on whether the participants grant approval when the first author contacts them with a request.
