# Supplementary material for: Developing a Virtual Reality Educational Tool to Stimulate Emotions for Learning: Focus Group Study
Source: JMIR Form Res. 2023 Mar 20;7:e41829. doi: 10.2196/41829 (PMC10131669; doi:10.2196/41829)
Supplement: Multimedia Appendix 1 [file formative_v7i1e41829_app1.docx]

Appendix I

**Questionnaire used to map students training needs (context): Development of a virtual practice arena with 360° videos and virtual reality technology (translated from Norwegian to English by the authors)**

In this project, we are developing a learning tool that uses 360° virtual reality videos. The videos will show realistic scenarios to enable students to observe and practice situations they might encounter in their future professional lives.

This survey has been sent to faculty and to second- and third-year students in the fields of nursing, social education, social work, and occupational therapy at XXX University, Norway, and to professionals in these fields who work with students in clinical placements. By completing this questionnaire, you will provide input on the types of situations we should include in the scripts for our 360° videos. Your input may contribute to new knowledge and increased quality in education. Participation is voluntary. By completing the questionnaire, you agree to participate.

Your responses are anonymous and will be handled confidentially. All data will be anonymized, and the results will not be linked to individual respondents. The data can provide a basis for publication internally and/or externally in a scientific journal.

Thank you for your contribution!

Questions from the online questionnaire; response options on a forced ranking scale

**1. To prepare you/your students for your/their future work, which of the following work/subject areas do you/your students need more experiences with?**

(Rank the areas in order of priority)

- Mental disorders
- Physical impairments
- Cognitive disabilities
- Drug addiction
- Collaboration with next-of-kin/caregivers
- Interdisciplinary collaboration
- Work with minorities
- Elderly care/geriatrics

**2. As professionals, we may encounter many different emotions in our clients/patients. To prepare you/your students for future professional practice, which CLIENT/PATIENT EMOTIONS would you/your students benefit from practice on?** (emotions as described by Sinding and Stiegler [20])

(Rank the emotions in order of priority)

- Joy/excitement
- Sadness/sorrow
- Fear/anxiety
- Anger
- Shame
- Guilt
- Disgust

**3. When we work professionally with people, some situations may trigger our own emotions. To prepare you/your students for future professional practice, which of the following of your OWN EMOTIONS do you/your students need more practice handling?** (emotions described by Sinding and Stiegler [20])

(Rank the emotions in order of priority)

- Joy/excitement
- Sadness/sorrow
- Fear/anxiety
- Anger
- Shame
- Guilt
- Disgust

**4. To better prepare you/your students for professional practice, which skills do you/your students need more practice in?**

- Relationships: Building trust
- Relationships: Boundary setting
- Relationships: Ending relationships
- Communication: Non-verbal communication
- Communication: Verbal communication
- Conflict management
- Interdisciplinary collaboration
- Collaboration with next-of-kin/caregivers
- User participation
- Cultural competence/cultural sensitivity
- Reflection on ethics, values, ​​and attitudes
- Coercion
- Professional writing (e.g., journal notes, reports, decision papers)
- Other
