# Supplementary material for: Developing a Virtual Reality Educational Tool to Stimulate Emotions for Learning: Focus Group Study
Source: JMIR Form Res. 2023 Mar 20;7:e41829. doi: 10.2196/41829 (PMC10131669; doi:10.2196/41829)
Supplement: Multimedia Appendix 2 [file formative_v7i1e41829_app2.docx]

Consolidated criteria for reporting qualitative studies (COREQ): 32-item checklist [27]

| **No** | **Item** | | **Question** | | | **Answer** | | | | **Reported in** |
| --- | --- | --- | --- | --- | --- | --- | --- | --- | --- | --- |
| **Domain 1: Research team and reflexivity** | | | | | | | | | |  |
| **Personal characteristics** | | | | | | | | | |  |
| 1 | Interviewer/facilitator | | Which author/s conducted the interview or focus group? | | | First author | | | | Data collection, page 7 |
| 2 | Credentials | | What were the researcher’s credentials? | | | First author: PhD  Co-authors 2&3: MSc  Last author: PhD | | | | Reported in author metadata |
| 3 | Occupation | | What was their occupation at the time of the study? | | | First author: Associate professor | | | | Reported in author metadata |
| 4 | Gender | | Was the researcher male or female? | | | Female | | | | Reported only by name |
| 5 | Experience and training | | What experience or training did the researcher have? | | | First author: Experienced  Co-authors:Experienced | | | | Page 7 and author metadata |
| **Relationship with participants** | | | | | | | | | |  |
| 6 | Relationship established | | Was a relationship established prior to study commencement? | | | Yes | | | | Page 6 (recruitment and participants), and page 12 (limitations) |
| 7 | Participant knowledge of the interviewer | | What did the participants know about the researcher? e.g. personal goals, reasons for doing the research | | | All participants knew the first author personally, and were informed about the reasons for doing the research both verbally and by the written information | | | | Page 12 (limitations) |
| 8 | Interviewer characteristics | | What characteristics were reported about the interviewer/facilitator? e.g. Bias, assumptions, reasons and interests in the research topic | | | Interviewer were the project leader, which all participants were aware of | | | | 12 |
| **Domain 2: Study design** | | | | | | | | |  | |
| **Theoretical framework** | | | | | | | | |  | |
| 9 | Methodological orientation and Theory | | What methodological orientation was stated to underpin the study? | | Qualitative interpretive approach (qualitative content analysis) | | | | Page 7, data-analysis | |
| **Participant selection** | | | | | | | | |  | |
| 10 | Sampling | | How were participants selected? | | | | Purposive sampling, recruited from project group | | Page 6 | |
| 11 | Method of approach | | How were participants approached? | | | | Asked verbally and in writing by project leader | | Page 6 | |
| 12 | Sample size | | How many participants were in the study? | | | | 12 | | Page 6 | |
| 13 | Non-participation | | How many people refused to participate or dropped out? Reasons? | | | | 6 | | Page 6 | |
| **Setting** | | | | | | | |  | | |
| 14 | | Setting of data-collection | | Where was the data collected? | | | 2 qualitative focus group interviews digitally at Zoom, and 1 at a meeting room at the university | Page 6-7 | | |
| 15 | | Presence of non-participants | | Was anyone else present besides the participants and researchers? | | | no | n/a | | |
| 16 | | Description of sample | | What are the important characteristics of the sample? | | | Project group members (faculty and students) | Page 6 | | |
| **Data collection** | | | | | | | |  | | |
| 17 | | Interview guide | | Were questions, prompts, guides provided by the authors? Was it pilot tested? | | | Interview guide was sent to informants in advance for preparation | n/a | | |
| 18 | | Repeat interviews | | Were repeat interviews carried out? If yes, how many? | | | No | n/a | | |
| 19 | | Audio/visual recording | | Did the research use audio or visual recording to collect the data? | | | Yes, audio recordings | Page 7 | | |
| 20 | | Field notes | | Were field notes made during and/or after the interview or focus group? | | | no | n/a | | |
| 21 | | Duration | | What was the duration of the interviews or focus group? | | | 60-70 minutes | Page 7 | | |
| 22 | | Data saturation | | Was data saturation discussed? | | | no | n/a | | |
| 23 | | Transcripts returned | | Were transcripts returned to participants for comment and/or correction? | | | Yes, follow-up questions to four participants | Page 7 | | |

| **Domain 3: analysis and findings** | | | |  |
| --- | --- | --- | --- | --- |
| **Data analysis** | | | |  |
| 24 | Number of data coders | How many data coders coded the data? | First author | Page 7-8 |
| 25 | Description of the coding tree | Did authors provide a description of the coding tree? | Written description in methods section | Not presented in article, but coding tree can be retrieved from authors |
| 26 | Derivation of themes | Were themes identified in advance or derived from the data? | Data-driven | Page 7 |
| 27 | Software | What software, if applicable, was used to manage the data? | Word | n/a |
| 28 | Participant checking | Did participants provide feedback on the findings? | Yes, comments | Page 7 |
| 29 | Quotations presented | Were participant quotations presented to illustrate the themes/findings? Was each quotation identified? e.g. participant number | Yes, quotations identified with “student” or “faculty”, but not participant/interview number to secure confidentiality | Results section page 8-10 |
| 30 | Data and findings consistent | Was there consistency between the data presented and the findings? | yes | Illustrated by presenting quotations |
| 31 | Clarity of major themes | Were major themes clearly presented in the findings? | Yes | Themes and categories are presented in findings, page 8-10 |
| 32 | Clarity of minor themes | Is there a description of diverse cases or discussion of minor themes? | yes |  |
